# Supplementary material for: The Clinical Application of Machine Learning-Based Models for Early Prediction of Hemorrhage in Trauma Intensive Care Units
Source: J Pers Med. 2022 Nov 14;12(11):1901. doi: 10.3390/jpm12111901 (PMC9699320; doi:10.3390/jpm12111901)
Supplement: Supplementary file 1 [file jpm-12-01901-s001.zip › JPM_supplementary_file_S1.pdf]

Supplementary file S1. Details of variables collected

I. Patient's profile

A. Age

B. Gender

II. Emergency department (ED) evaluation and initial treatment

A. Labeled as "Trauma team activation" or not

Trauma team activation criteria:

(a)Unstable vital signs:

1. Unstable blood pressure: Systolic blood pressure < 90mmHg
2. Consciousness disturbance: Glasgow Coma Scale <13
3. Respiratory failure:Respiratory rate > 30 times/ minutes or < 10 times/minutes,Pulse oximeter< 90%

(b)Major trauma mechanism

1. Fall from > 6 meters height or above the second floor
2. Crush injury by heavy objects
3. Be thrown out of the vehicle
4. Casualty noted of same vehicle passengers

(c) High risk injury site

1. Penetration wound at head, neck, or trunk area
2. At least second-degree burn injury on the face

(d)Initiate as attending doctor's judgment

1. Pedestrian hit by motor/vehicle
2. Persons with multiple trauma and age <10 or > 65 years old
3. Unstable or complicated pelvic fracture
4. Proximal long bone(Humerus bone or Femur bone) fracture
5. Traumatic amputation above the wrist or ankle
6. Obvious flail chest
9. Limbs paralysis

**Trauma team activation criteria is cited from the Annual report of the trauma center, Chang Gung Memorial Hospital,2017. (Could be accessed by:https://cghdpt.cgmh.org.tw/files/downloads/0617bc56-acde-4ba3-8537-d7aae883f74b.pdf)

B. Abbreviated Injury Scale(AIS)

The Abbreviated Injury Scale (AIS) is an anatomically-based injury severity scoring system that classifies each injury by body region on a 6 point scale. AIS is the system used to determine the Injury Severity Score (ISS) of a multiply injured patient.

The AIS classifies individual injuries by body region as follows:

- AIS 1 – Minor
- AIS 2 – Moderate
- AIS 3 – Serious
- AIS 4 – Severe
- AIS 5 – Critical
- AIS 6 – Maximal (currently untreatable)

Reference: Abbreviated Injury Scale (AIS) - Overview [Internet]. Association for the Advancement of Automotive Medicine. 2019 [cited 7 August 2019]. Available from: https://www.aaam.org/abbreviated-injury-scale-ais/

C. Injury severity score(ISS)

** The ISS is calculated as the sum of the squares of the highest AIS code in each of the three most severely injured ISS body regions. These body regions are:

- Head or neck
- Face
- Chest
- Abdominal or pelvic contents
- Extremities or pelvic girdle
- External

Injury Severity Scores range from 1 to 75. If an injury is assigned an AIS of 6 (identifying a currently untreatable injury), the ISS score is automatically assigned 75.

D. Intubation for the endotracheal tube was done at ED or not

E. Cardiopulmonary resuscitation (CPR) was done at ED or not

F. Received transarterial embolization(TAE) at ED or not

G. Glasgow Coma Score(GCS) at ED, including timing at arriving at ED and leaving ED

Glasgow Coma Score(GCS):

The Glasgow Coma Scale (GCS) is used to objectively describe the extent of impaired consciousness in all types of acute medical and trauma patients.

The total Coma Score thus has values between three and 15, three being the worst and 15 being the highest.

Eye Opening Response

- Spontaneous--open with blinking at baseline 4 points
- To verbal stimuli, command, speech 3 points
- To pain only (not applied to face) 2 points

- No response 1 point

Verbal Response

- Oriented 5 points
- Confused conversation, but able to answer questions 4 points
- Inappropriate words 3 points
- Incomprehensible speech 2 points
- No response 1 point

Motor Response

- Obeys commands for movement 6 points
- Purposeful movement to painful stimulus 5 points
- Withdraws in response to pain 4 points
- Flexion in response to pain (decorticate posturing) 3 points
- Extension response in response to pain (decerebrate posturing) 2 points
- No response 1 point

References Teasdale G, Jennett B. Assessment of coma and impaired consciousness. Lancet 1974; 81-84. Teasdale G, Jennett B. Assessment and prognosis of coma after head injury. Acta Neurochir 1976; 34:45-55.

III. Vital signs at intensive care unit(ICU)

A. Systolic blood pressure and diastolic blood pressure(mmHg)

B. Heart rate(Beats/minutes)

C. Pulse oximeter(%)

D. Respiratory rate(Times/minutes)

III. Laboratory findings at intensive care unit(ICU)

A. Hematology

1. White Blood Cell(1000/uL)
2. Hemoglobin(g/dL)
3. Hematocrit(%)
4. Mean corpuscular volume(MCV)
5. Platelets(1000/dL)
6. Prothrombin time(Seconds)
7. International normalized ratio(INR)
8. Activated Partial Thromboplastin Time(APTT, Seconds)
9. Fibrinogen(mg/dL)

B. Biochemistry

1. Blood urea nitrogen(BUN,mg/dL)
2. Creatinine(mg/dL)

3. Sodium(Na,mEq/L)
4. Potassium(K,mEq/L)
5. Calcium(Ca,mEq/L)
6. Chloride(Cl,mEq/L)
7. Magnesium(Mg,mEq/L)
8. Lactate(mg/dL)
9. Amylase(U/L)
10. Lipase(U/L)
11. Total bilirubin(mg/dL)
12. Alkaline Phosphatase(Alk-P, U/L)
13. Aspartate AminoTransferase(AST,U/L)
14. Alanine Aminotransferase(ALT, U/L)
15. Blood Sugar(mg/dL)
16. Albumin(g/dL)
17. Procalcitonin(ng/mL)

C. Arterial blood gas

1. Temperature(°C)
2. pH
3. paCO2(mmHg)
4. paO2(mmHg)
5. Saturation(%)
6. standard base excess (SBE,mmol/L)
7. Bicarbonate(HCO3,mmol/L).
