# Supplementary material for: The Clinical Application of Machine Learning-Based Models for Early Prediction of Hemorrhage in Trauma Intensive Care Units
Source: J Pers Med. 2022 Nov 14;12(11):1901. doi: 10.3390/jpm12111901 (PMC9699320; doi:10.3390/jpm12111901)
Supplement: Supplementary file 1 [file jpm-12-01901-s001.zip › JPM_supplementary_table_S1.pdf]

Supplementary Table S1. International Classification of Diseases Clinical Modification (ICD-CM) codes used for diagnosis, associated injury, underlying disease, and complications in the current study.

Diagnosis	ICD-9-CM codes	ICD-10-CM codes
Inclusion criteria (torsal trauma)		
Traumatic pneumothorax and hemothorax	860	S270(XXA,XXD,XXS)<br>S271(XXA,XXD,XXS)<br>S272 (XXA,XXD,XXS)<br>S21309(A,D,S)
Injury to heart and lung	861	S26(XXXA,XXXD,XXXS)
Injury to other and unspecified intrathoracic organs	862	S273(XXA,XXD,XXS)
Injury to chest wall	922	S200(0-2)<br>S20219(A,D,S)<br>S20229(A,D,S)<br>S2020 (XA,XD,XS)
Injury to gastrointestinal tract	863	S3630(XA,XD,XS)<br>S36400(A,D,S)<br>S36408(A,D,S)<br>S36409(A,D,S)
Injury to liver	864	s361 (1,2,3)
Injury to spleen	865	S360 (0,2,3,9)
Injury to kidney	866	S307(0-6,9)
Injury to pelvic organs	867	S3760(XA,XD,XS)<br>S37409 (A,D,S)<br>S37509 (A,D,S)<br>S37892 (A,D,S)<br>S37889 (A,D,S)<br>S3790 (A,D,S)

Injury to other intra-abdominal organs	868	S3720(XA,XD,XS)<br>S3730 (XA,XD,XS)<br>S3790 (A,D,S)<br>S37819 (A,D,S)<br>S36899 (A,D,S)
Abdominal wall and back injury	922	S31609(A,D,S)<br>S31001(A,D,S)<br>S3681 (XA,XD,XS)<br>S301 (XXA,XXD,XXS)<br>S300 (XXA,XXD,XXS)<br>s3020(1,2)
**Exclusion criteria**<br>(brain hemorrhage)		
Subarachnoid, subdural, and extradural hemorrhage	852<br>430<br>431	S066 (X0-X9)<br>S065(X0-X9)<br>S064(X0-X9)
Other and unspecified intracranial hemorrhage	432	S0190(XA,XD,XS)
